# PM2.5 Induces Cardiomyoblast Senescence via AhR-Mediated Oxidative Stress

**DOI:** 10.3390/antiox13070786

**Published:** 2024-06-28

**Authors:** Tiantian Liu, Bin Jiang, Baoqiang Fu, Changyi Shang, Haobin Feng, Tao Chen, Yan Jiang

**Affiliations:** 1 School of Biology and Basic Medic Sciences, Suzhou Medical College, Soochow University, Suzhou 215123, Chinashangchangyi187@163.com (C.S.); hbfeng@stu.suda.edu.cn (H.F.); 2 The First Affiliated Hospital of Soochow University, Suzhou 215005, China; jbin@suda.edu.cn; 3 MOE Key Laboratory of Geriatric Disease and Immunology, Soochow University, Suzhou 215123, China; 4 Jiangsu Key Laboratory of Preventive and Translational Medicine for Major Chronic Non-Communicable Diseases, Soochow University, Suzhou 215123, China; 5 School of Public Health, Suzhou Medical College, Soochow University, Suzhou 215123, China

**Keywords:** PM2.5, cardiomyoblast cells, senescence, AhR, oxidative stress

## Abstract

Previous research has established a correlation between PM2.5 exposure and aging-related cardiovascular diseases, primarily in blood vessels. However, the impact of PM2.5 on cardiomyocyte aging remains unclear. In this study, we observed that extractable organic matter (EOM) from PM2.5 exposure led to cellular senescence in H9c2 cardiomyoblast cells, as characterized by an increase in the percentage of β-galactosidase-positive cells, elevated expression levels of p16 and p21, and enhanced H3K9me3 foci. EOM also induced cell cycle arrest at the G1/S stage, accompanied by downregulation of CDK4 and Cyclin D1. Furthermore, EOM exposure led to a significant elevation in intracellular reactive oxygen species (ROS), mitochondrial ROS, and DNA damage. Supplementation with the antioxidant NAC effectively attenuated EOM-induced cardiac senescence. Our findings also revealed that exposure to EOM activated the aryl hydrocarbon receptor (AhR) signaling pathway, as evidenced by AhR translocation to the nucleus and upregulation of Cyp1a1 and Cyp1b1. Importantly, the AhR antagonist CH223191 effectively mitigated EOM-induced oxidative stress and cellular senescence. In conclusion, our results indicate that PM2.5-induced AhR activation leads to oxidative stress, DNA damage, and cell cycle arrest, leading to cardiac senescence. Targeting the AhR/ROS axis might be a promising therapeutic strategy for combating PM2.5-induced cardiac aging.

## 1. Introduction

Ambient fine particle matter, commonly known as PM2.5 (particles with an aerodynamic diameter of less than 2.5 μm), has emerged as the world’s heaviest health burden [1]. It is estimated that approximately 90% of the population still reside in areas where PM2.5 concentrations surpass the guideline set by the World Health Organization [2]. The toxicity of PM2.5 is largely influenced by its complex composition, with particular attention being given to its organic components due to their high concentration of harmful substances [3]. Recent studies have linked the organic components of PM2.5 to the development of ferroptosis [4]. Our previous research revealed that the extractable organic matter (EOM) from PM2.5 causes heart malformation and dysfunction in zebrafish [5]. Therefore, further investigation is required to fully understand the cardiac toxicity of EOM.

Cardiac aging serves as a major risk factor for cardiovascular disease, the leading cause of human mortality [6]. The impact of PM2.5 exposure on cardiac health is of particular concern in older adults, as they are more susceptible to its negative effects. Studies have shown that PM2.5 exposure can impair vasomotor function and heart rate variability in this specific population subset [7]. In elderly individuals, studies have linked PM2.5 exposure to increased corrected QT duration and cardiac repolarization [8,9]. Moreover, recent evidence indicates that PM2.5 can accelerate aging in the skin and nervous system [10,11]. Despite these findings, the direct influence of PM2.5 exposure on cardiac aging and the underlying mechanisms involved have yet to be thoroughly investigated.

Myocardial cell senescence, which is influenced by genetic predisposition, lifestyle factors, and environmental factors, plays a crucial role in tissue remodeling during wound repair and embryogenesis [12]. However, maladaptive cardiac senescence has been associated with reduced heart function and aging-related cardiovascular diseases, such as cardiomyopathies and arrhythmias [13,14]. While existing research on PM2.5-induced cardiovascular diseases primarily focuses on cardiomyocyte apoptosis [15], there is limited insight into how the heart responds to environmental stress prior to the onset of apoptosis. Cellular senescence is posited as an antecedent to cellular apoptosis, either preceding the initiation of apoptosis or exerting its influence on adjacent cells via the release of cytokines [16]. Therefore, it is imperative to explore the effect of PM2.5 on cardiomyocytes by investigating cardiac senescence and its related mechanisms.

We have previously reported that EOM from PM2.5 can lead to cardiac defects via the activation of the aryl hydrocarbon receptor (AhR) signaling pathway [17]. AhR functions as an environmental sensor that can be agonized by various environmental pollutants such as dioxins. When AhR binds to these ligands, it translocates to the nucleus and enhances the transcriptional activation of target genes, including cytochrome P450 family 1(CYP1s) [18]. The main function of the P450 family is to facilitate the detoxification and elimination of various xenobiotic compounds such as drugs and pollutants. Emerging evidence suggests that CYP1s can also influence reactive oxygen species (ROS) production and mitochondrial function [19]. Oxidative stress is a well-known contributor to cardiac diseases, characterized by an imbalance between generation of ROS and the redox state [20]. Excessive levels of ROS can cause harm by inducing DNA damage, protein oxidation, lipid peroxidation, and disruption of cellular homeostasis. These molecular disruptions trigger cellular senescence, a state of irreversible growth arrest, thus contributing to tissue dysfunction and age-related pathologies [21]. Moreover, ROS have also been found to activate signaling pathways involved in cellular senescence, such as the p53-p21 and p16-Rb pathways. Additionally, ROS can promote the secretion of pro-inflammatory cytokines, which are implicated in age-related cardiovascular disease [22].

As a downstream target of AhR, CYP1 enzymes, such as CYP1A1 and CYP1B1, can generate ROS as a byproduct during their enzymatic reactions [23]. In our previous study, we have also found evidence of ROS overproduction mediated by the AhR in cardiac defects induced by exposure to PM2.5 [24]. Building upon these findings, we propose the hypothesis that exposure to PM2.5 activates AhR signaling pathway, leading to oxidative stress and ultimately contributing to cardiac aging. Roles of oxidative stress and AhR in this process were investigated to explore the underlying mechanisms.

## 2. Materials and Methods

### 2.1. Chemicals

N-Acetyl-L-cysteine (NAC, CAS number 616-91-1) was obtained from Aladdin Chemicals (Shanghai, China). CH223191 was purchased from MedChemExpress (Shanghai, China). All other chemicals were obtained from Sinopharm Chemical Reagent Co. Ltd. (Shanghai, China) unless otherwise specified.

### 2.2. Collection of PM2.5 and EOM Purification

As we previously described, PM2.5 was gathered in an urban area of Suzhou, China, during the winter season [17]. In brief, samples were collected on 47 mm quartz films for 24 h each day with a Tianhong TH-150C PM2.5 sampler (Wuhan, China). The films were baked in a muffle furnace for 2 h at 500 °C before PM2.5 collection and subjected to Soxhlet extraction using dichloromethane. The extracted samples were concentrated with a rotary evaporator and nitrogen blowing apparatus before being dissolved in DMSO. EOMs were then combined and kept at −80 °C.

### 2.3. Culture of the H9c2 Rat Cardiomyoblasts

The rat embryonic cardiac cell line H9c2 cardiomyoblast was purchased from Cell Bank of Chinese Academy of Sciences (Shanghai, China). H9c2 cardiomyoblasts were maintained following established protocols as reported previously [25,26]. In brief, the cells were cultivated in a 5% CO_2_ atmosphere at 37 °C, using DMEM supplemented with fetal bovine serum (10%, Thermo Fisher Scientific, Waltham, MA, USA) and penicillin-streptomycin (100 U/mL, Thermo Fisher Scientific). Cells were treated with EOM (10 μg/mL) for 48 h with or without prior incubation with CH or NAC.

### 2.4. Cell Viability Determination by MTT Assay

H9c2 cardiomyoblasts cells were plated into a 96-well plate. Subsequently, they were treated with varying concentrations of EOM (3, 10, 30, and 50 μg/mL) for a duration of 48 h. A negative control using DMSO (0.1%) as the vehicle and a positive control using Triton X-100 (0.5%) were also included in the experiment. Following this, a solution of MTT (MTT: Boster, AR1156) was introduced and incubated for 4 h. Subsequently, Formanzan solution was added and an additional incubation was performed for 4 h. With the microplate reader (Molecular Devices, Sunnydale, CA, USA), the changes in absorbance at 570 nm were measured. The outcomes are presented as a relative percentage compared to the control cells.

### 2.5. Apoptosis Analysis by Flow Cytometry via Annexin V/Propidium Iodide Staining

Cell apoptosis was examined via an Annexin V-FITC/Propidium iodide assay. Initially, H9c2 cardiomyoblasts were collected and suspended at a concentration of 1 × 10^5^ cells in 200 µL of Annexin V binding buffer. Subsequently, the cells were incubated with 4 µL of 0.5 mg/mL PI and 2 µL of Annexin V-FITC at room temperature for 15 min in the dark. Flow cytometry (FACS Calibur, BD Biosciences, Mountain View, CA, USA) was utilized to detect the signal, and the data were processed using software FlowJo vX.0.7 (FlowJo LLC, Ashland, OR, USA).

### 2.6. Western Blotting

Cells were harvested, then lysed in a buffer containing protease inhibitors and phosphatase inhibitors. The lysates were then incubated on ice for half an hour, followed by centrifugation (20 min, 10,000 rpm, 4 °C) to clear the lysates. The Bradford method was used to assess the protein concentration. Afterward, protein with equal quantities (20 μg) were loaded, separated by SDS-PAGE, and transferred onto a membrane. The membrane was then exposed to primary antibodies for probing. The primary antibodies utilized consisted of anti-PCNA, anti-CyclinE, anti-CyclinD1, anti-CDK4, and anti-CDK2 (all from Santa Cruz Biotechnology, Santa Cruz, CA, USA). Following this, the membranes underwent incubation with a secondary antibody conjugated to horseradish peroxidase (MultiSciences Biotech, Hangzhou, China). Protein bands were then visualized by enhanced chemiluminescent staining (Amersham Biosciences, Amersham, UK) and image analysis software was used to quantify.

### 2.7. EdU Cell Incorporation Assay

After treating the cells with EOM for 48 h, the EdU solution was introduced and incubated for 2 h at 37 °C. Subsequently, the cells were treated with 4% paraformaldehyde for 30 min for fixation, followed by a 5-min treatment with glycine (2 mg/mL, Solarbio, Beijing, China). Next, the cells were treated with 0.5% Triton X-100 for 10 min to achieve permeabilization. The cells were incubated in the dark with Apollo Reaction Solution, following the instructions provided in the RiboBio EdU cell proliferation detection kit (RiboBio, Guangzhou, China). Finally, the nuclei were counterstained with DAPI (Invitrogen, Carlsbad, CA, USA), and fluorescent images were captured using a fluorescence microscope (Olympus IX73, Tokyo, Japan).

### 2.8. Determination of Cell Cycle Distribution

For the tested samples, 1 × 10^6^ cells were collected and fixed with 70% ethanol at 4 °C for 2 h. Then, the cells were cleared and re-suspended in PBS. The DNA was stained with propidium iodide)/RNaseA (both from Sigma-Aldrich, St. Louis, MO, USA) solution supplemented with 0.2% Triton X-100 for 30 min at room temperature in the dark. Red fluorescence and light scattering were obtained using flow cytometry (FACSCalibur BD Biosciences) and cell cycle distribution patterns were analyzed by FlowJo vX.0.7 (FlowJo LLC).

### 2.9. Immunofluorescence Staining

For immunofluorescence analysis, the cells were fixed for 15 min in 4% paraformaldehyde. Following permeabilization with 0.3% triton X-100 for 1 h, the cells underwent blocking with 10% BSA for 30 min at 37 °C before incubation with primary antibody overnight at 4 °C. The primary antibodies utilized in this research were anti-AhR (Santa Cruz, CA, USA), anti-P21(Abcam, Cambridge, UK), γ-H2a.X (Abcam), and anti-H3K9me3 (Abcam). Subsequently, the cells were probed with fluorescence-conjugated secondary antibody (Invitrogen) at room temperature for 1 h. Prior to the final examination, the cell nuclei were stained with DAPI (Abcam) for 5 min. The fluorescent images were captured using an Olympus microscope (Olympus, Tokyo, Japan).

### 2.10. RNA Extraction, cDNA Synthesis, and qPCR Reactions

The samples were subjected to TRIzol reagent (Tiangen, Beijing, China) to extract total RNA. Subsequently, 1 μg of total RNA was employed as a template for first-strand cDNA following the manufacturer’s instructions of the RevertAid™ First Strand cDNA Synthesis Kit (Thermo Scientific). Quantitative PCR was carried out on an ABI QuantStudio 6 qPCR system (Applied Biosystems, Foster City, MA, USA) with SYBR Green qPCR Master Mix (Thermo Scientific). The qPCR protocol consisted of an initial denaturation at 95 °C for 2 min, followed by 40 cycles (95 °C, 10 s; 60 °C, 30 s; and 70 °C, 45 s). The primer sequences are detailed in Table S1, with β-actin serving as the reference gene. The relative expression levels of target genes were determined using the 2^−ΔΔct^ method.

### 2.11. β-Galactosidase Staining

The cells were fixed with fixative solution (Beyotime, Shanghai, China) at room temperature for 15 min. Following PBS washing, the cells were incubated with β-galactosidase staining solution (Beyotime) overnight in a dry incubator (37 °C, no CO_2_). Images were captured with an inverted microscope (200×) after proper washing with PBS. The quantification of senescent cells was conducted by visually assessing the presence of blue-stained cells, with at least 6 fields of view documented for each group.

### 2.12. Mitochondrial ROS (mtROS) and Intracellular ROS Detection

To assess mtROS levels and intracellular ROS levels, MitoSOX™ Red (0.5 µM, ThermoFisher) and DCFH-DA (10 µM, Beyotime) were added to living cells and incubated at 37 °C for 20 min in darkness. Subsequently, the labeled cells were fixed with 4% PFA, and counterstained with DAPI. The images were obtained using a confocal microscope (Carl Zeiss, Oberkochen, Germany).

### 2.13. Statistical Analysis

All experimental data were performed individually at least three times, and the results are presented as mean ± SD (standard deviation) and were analyzed using Graph Pad Prism 5 software. To determine the significance of differences between groups, appropriate statistical tests were employed based on the number of groups being compared. For comparisons between two groups, a Student’s *t*-test was utilized. For comparison involving more than two groups, a one-way ANOVA was performed, followed by Turkey’s multiple comparison test. A *p*-value below 0.05 was considered statistically significant.

## 3. Results

### 3.1. Effects of EOM on Cell Viability and Morphology

**Figure 1:**
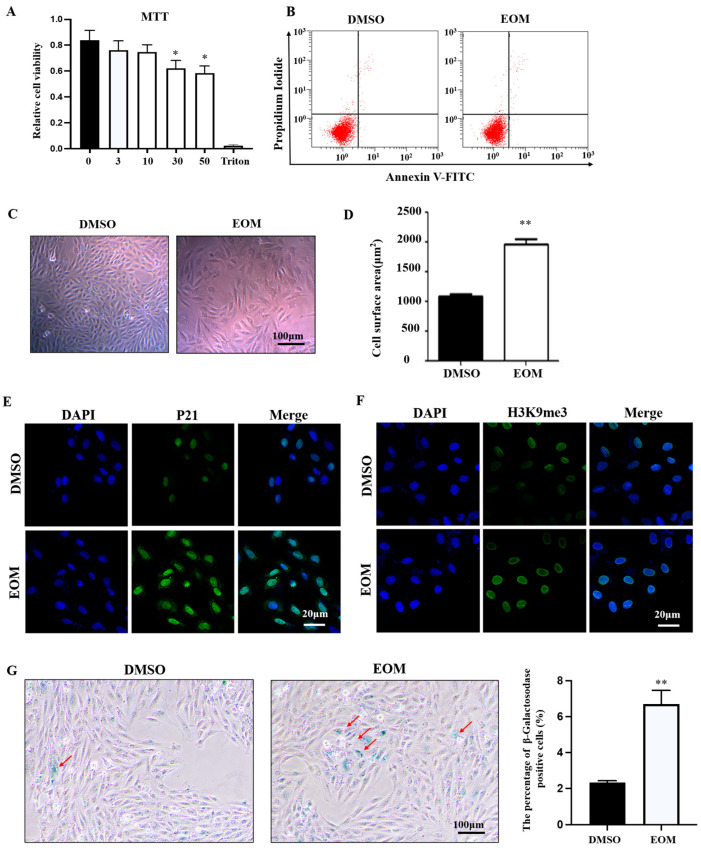
Effects of EOM on viability and morphology of H9c2 cardiomyoblasts (n ≥ 4). (**A**) Viability assessment by MTT assay. 0, 3, 10, 30, 50: EOM concentrations (μg/mL); (**B**) Apoptosis assessed by flow cytometry; (**C**) Representative morphological images. Scale bar: 100 μm; (**D**) Quantification of cell size by measuring the cell surface area using Image J software (Version: 1.53e) analysis; (**E**,**F**) Exemplary immunofluorescence images display p21 and H3K9me3 staining, with DAPI used for nuclear counterstaining. Scale bar: 20 μm; (**G**) Illustrative images depicting senescence-associated β-galactosidase staining in H9c2 cells. Senescent cells are highlighted in blue and marked with red arrows. Scale bar: 100 μm. The graph illustrates the proportion of β-galactosidase-positive cells, with data obtained and evaluated from at least six randomly chosen fields. EOM: 10 μg/mL, * *p* < 0.05, ** *p* < 0.01.

Cell viability was assessed following exposure to various concentrations of EOM (0 μg/mL, 3 μg/mL, 10 μg/mL, 30 μg/mL, 50 μg/mL) for a duration of 48 h, with Triton X-100 used as a positive control. The results revealed a significant decrease in cell viability in the EOM groups with concentrations exceeding 10 μg/mL (Figure 1A). Flow cytometry analysis showed that EOM at 10 μg/mL did not induce apoptosis in H9c2 cells (Figure 1B). Thus, a concentration of 10 μg/mL was selected for subsequent experiments.

As shown in Figure 1C, EOM-exposed cells exhibited a flattened morphology with distinguishable nuclei. Significantly, cells within the EOM-treated group exhibited larger surface areas compared to those in the control group (Figure 1D). Furthermore, exposure to EOM resulted in upregulation of cellular senescence biomarkers p21 and H3K9me3 (Figure 1E,F). Meanwhile, there was a notable increase in the number of cells showing positive β-galactosidase staining in the EOM-treated group compared to the controls (Figure 1G). Therefore, these findings indicate that the exposure of EOM derived from PM2.5 induces cellular aging without impacting apoptosis.

### 3.2. EOM Exposure Induces Cell Cycle Arrest

**Figure 2:**
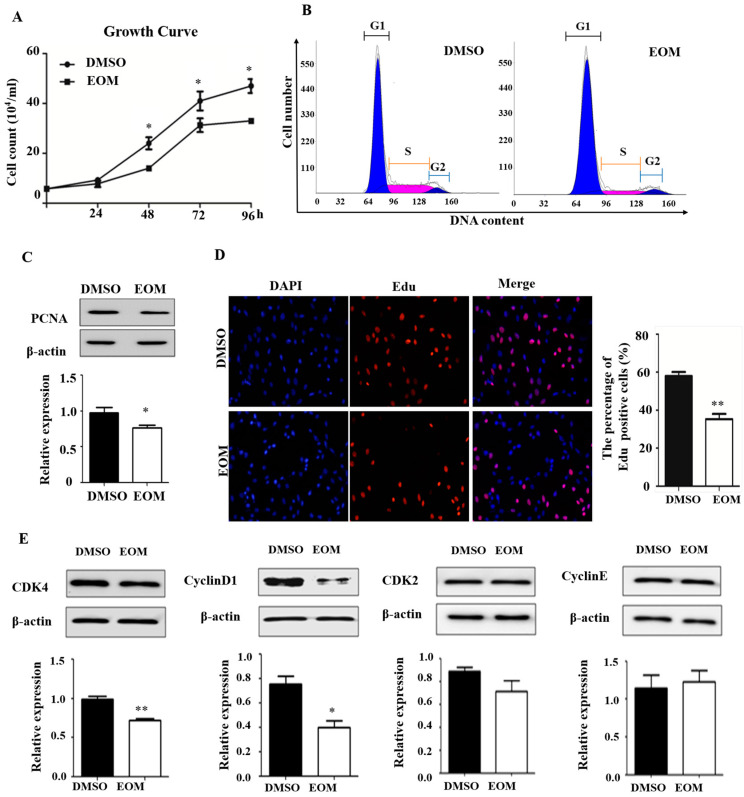
Effects of EOM exposure on the cell cycle (n ≥ 3). (**A**) Cell growth curves of H9c2 cardiomyoblasts cultured with or without EOM for 4 consecutive days; (**B**) Cell cycle analysis by flow cytometry; (**C**) Protein expression levels of PCNA; (**D**) EdU incorporation assay utilized to evaluate DNA synthesis. Red EdU staining spots indicate cells undergoing DNA synthesis, and blue DAPI staining highlights nuclei. The graph illustrates the percentage of EdU-positive cells relative to the total DAPI-stained cells, with mean values ± SE indicated. Scale bar: 100 μm; (**E**) Protein expression levels of CDK4, cyclin D1, CDK2, and cyclin E. EOM: 10 μg/mL, * *p* < 0.05, ** *p* < 0.01.

As demonstrated in Figure 2A, EOM exposure significantly inhibited cell growth in H9c2 cardiomyoblasts. Flow cytometry analysis showed that EOM notably decreased the proportion of cells in S-phase compared to the control group (11.74 ± 0.35% vs. 24.19 ± 1.25%, *p* < 0.05, Figure 2B). Conversely, there was a considerable increase in the percentage of cells in G1 phase, from 72.42 ± 2.66% (control) to 83.50 ± 0.9% (EOM). Furthermore, our results indicated that EOM treatment led to a reduction in the protein expression levels of PCNA, which serves as a proliferation marker (Figure 2C). Additionally, the results obtained from the EdU incorporation assay revealed a significant decrease in the percentage of EdU-positive cells following EOM treatment compared to the control group (35 ± 4.9% vs. 58 ± 3.0%, *p* < 0.01, Figure 2D). Although no significant differences were observed in CDK2 and Cyclin E expression levels, exposure to EOM resulted in a significant downregulation of CDK4 and Cyclin D1 protein levels (*p* < 0.01, *p* < 0.05, Figure 2E). Taken together, these results suggest that after 48 h of EOM exposure, the cell cycle of H9c2 cardiomyoblasts was arrested at the G1 phase.

### 3.3. Oxidative Stress Mediates EOM-Induced Cardiac Senescence

**Figure 3:**
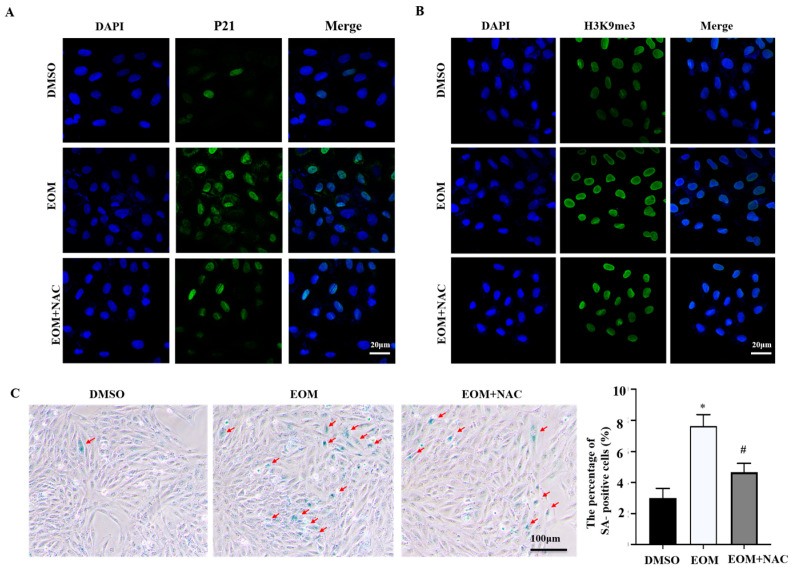
Oxidative stress was involved in EOM-induced cardiac senescence in H9c2 cardiomyoblasts (n ≥ 3). (**A**,**B**) Exemplary images of immunofluorescence staining for p21 and H3K9me3 in H9c2 cells after treatment with vehicle or EOM, with or without NAC, with green indicating p21 and H3K9me3, blue representing the nucleus, DAPI. Scale: 20 μm; (**C**) Representative image of β-gal–positive staining; cells from 6 fields of view were randomly counted, red arrow indicates senescent cells. Scale: 100 μm. EOM: 10 μg/mL, NAC: 5 nM; *: *p* < 0.05, compared to the DMSO group. #: *p* < 0.05 compared to the EOM group.

The immunofluorescence staining data depicted in Figure 3A demonstrate elevated levels of p21 expression in EOM-treated cells compared to the control group. However, this effect was mitigated by the administration of N-acetylcysteine (NAC). NAC also reversed the enhanced H3K9me3 staining induced by EOM, as shown in Figure 3B. Moreover, NAC countered the increase in β-galactosidase-positive cells triggered by EOM, depicted in Figure 3C. Taken together, these findings indicate that oxidative stress plays a critical role in EOM-induced cardiac senescence.

### 3.4. EOM Exposure Activates the AhR Signaling Pathway and Mediated Oxidative Stress

**Figure 4:**
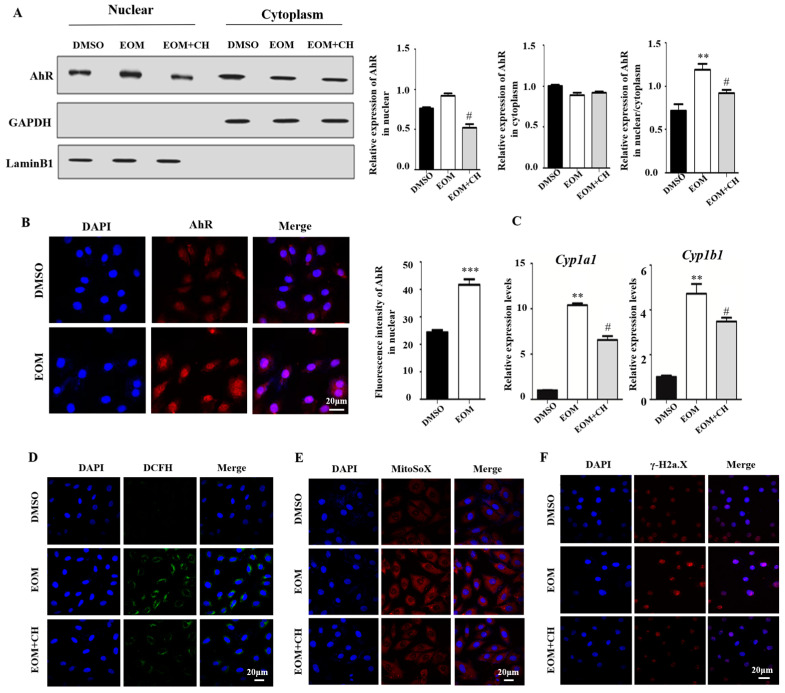
AhR is activated and mediates EOM-induced oxidative stress and DNA damage in H9c2 cardiomyoblasts (n ≥ 3). (**A**) Cytoplasm proteins and nuclear proteins were isolated and subjected to AhR expression analysis via Western blot. Loadings were normalized against GAPDH (cytoplasm) and LaminB1 (nuclear), respectively; (**B**) Immunofluorescence staining of AhR. The graph illustrates the quantification of relative fluorescence intensity of AhR in the nuclear; (**C**) mRNA expression levels of Cyp1a1 and Cyp1b1; (**D**,**E**) Intracellular ROS and mtROS levels assessed by staining with DCFH-DA or MitoSox; (**F**) γ-H2a.X staining. Scale bar: 20 μm. EOM:10 μg/mL; CH: CH223191 at 3 μg/mL. **: *p* < 0.01, ***: *p* < 0.001, compared to the DMSO group. #: *p* < 0.05, compared to the EOM group.

We then demonstrated that in the EOM group, nuclear fraction had a higher level of AhR compared to the cytoplasm, indicating that AhR is predominantly localized in the nucleus after EOM treatment (Figure 4A). The immunofluorescence experiment confirmed stronger AhR nucleus staining in the EOM-treated group than in the control group (*p* < 0.001, Figure 4B). Quantitative PCR results showed a significant upregulation of AhR downstream genes Cyp1a1 and Cyp1b1 in response to EOM treatment (10-fold and 4-fold change, respectively, Figure 4C). Notably, the addition of the AhR antagonist CH effectively inhibited the effects of EOM on AhR nuclear transport and the overexpression of Cyp1a1 and Cyp1b1 (Figure 4A–C).

In addition, EOM treatment significantly increased the levels of intracellular ROS and mtROS compared to the control group (Figure 4D,E). Furthermore, EOM exposure resulted in DNA double-strand breaks, as indicated by enhanced γ-H2a.X staining signals (Figure 4F). Supplementation with CH aborted the EOM-induced intracellular ROS/mtROS overgeneration and DNA double-strand breaks. These findings suggest that EOM exposure activates AhR signaling, leading to oxidative stress and DNA double strand breaks in H9c2 cells (Figure 4D–F).

### 3.5. AhR Mediates EOM-Induced H9c2 Senescence

**Figure 5:**
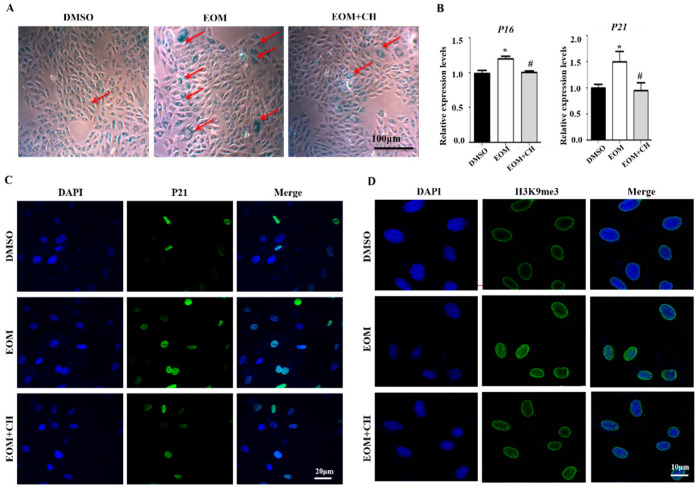
AhR activation is involved in EOM-induced H9C2 senescence (n ≥ 3). (**A**) Exemplary images showing senescence-associated β-galactosidase staining in H9c2 cardiomyoblasts. Senescent cells are indicated by red arrows and stained blue. Scale: 100 μm; (**B**) mRNA expression levels of p16 and p21; (**C**) Illustrative immunofluorescence images displaying p21 staining in green, with nuclear staining DAPI in blue. Scale: 20 μm; (**D**) Representative immunofluorescence images displaying the heterochromatin foci marker H3K9me3 (green) with nuclear staining using DAPI (blue). Scale: 10 μm. EOM:10 μg/mL; CH: CH223191 at 3 μg/mL; *: *p* < 0.05, compared to the DMSO group. #, *p* < 0.05, compared to the EOM group.

We found that the addition of AhR inhibitor CH attenuated the EOM-induced increase in the proportion of β-galactosidase-positive cells (Figure 5A) and mRNA overexpression of p16 and p21 (*p* < 0.05, Figure 5B). EOM-upregulated protein expression levels of p21 were also attenuated by CH (Figure 5C). Additionally, CH mitigated the pronounced staining signals of H3K9me3 induced by EOM (Figure 5D). These findings suggest that AhR plays an important role in EOM-induced cardiac senescence.

## 4. Discussion

According to the World Health Organization, the number of individuals aged 60 and above is projected to double by 2050, and cardiovascular disease continues to be the leading cause of death among older adults [27]. There is a substantial body of research indicating that exposure to PM2.5 is associated with cardiovascular morbidity and mortality [28]. Additionally, evidence suggests that PM2.5 exposure contributes to skin aging [10] and accelerates cognitive decline [29]. However, a comprehensive investigation into the impact of PM2.5 on cardiac aging and its associated mechanisms has been lacking. Our current study presents compelling evidence, marking the first report on the direct aging effect of PM2.5 on cardiac cells, specifically rat cardiomyoblasts. Our findings demonstrate that exposure to PM2.5-derived EOM incites cellular senescence via the mediation of AhR-induced oxidative stress.

Cellular senescence is a well-known biological process that is primarily activated by cell cycle signal pathways, such as p53-p21 and p16-Rb pathways [30]. Previous research has established a link between PM2.5 exposure and abnormal cell proliferation in human buccal cells and umbilical vein endothelial cells [31,32]. In this study, we found that EOM from PM2.5 increased the expression levels of p21 and 16, thereby interfering with the proliferation of H9c2 cells. Further analysis revealed that EOM induced cell cycle arrest at the G1 stage via downregulating the expression of Cyclin D1 and CDK4 (Figure 2). Consistent with our findings, Yuan et al. reported growth retardation and G0/G1 phase arrest in rat embryos upon PM2.5 exposure [33]. Fascinatingly, while previous studies have demonstrated that PM2.5 exposure leads to cardiac apoptosis in zebrafish and mice [5,34], our current investigation did not reveal a substantial rise in apoptosis (Figure 1). We hypothesize that this disparity, beyond potential species-specific distinctions, may stem from differences in the intensity and duration of exposure. Although cellular senescence and apoptosis are distinct cell processes, they can intersect under certain circumstances. Cells may undergo apoptosis in response to prolonged senescence [16]. In our study, we specifically examined the impact of EOM in the early stages before apoptosis was initiated. Further research is required to comprehensively elucidate the connection between senescence and apoptosis induced by environmental hazards.

It is widely recognized that the accumulation of senescent cells contributes to accelerated aging and dysfunction in organisms [35]. Cardiomyocytes, accounting for approximately 80% of the cellular volume and 30–40% of the total cell population in the heart, play a crucial role in maintaining normal heart function. Given their significance in sustaining heart function, the senescence of cardiomyocytes can have profound implications [36]. Our present study is the first to demonstrate that EOM exposure induces cellular senescence in cardiomyoblasts characterized by upregulation of aging-related genes (p16 and p21), chromatin structure remodeling (H3K9me3), and increased β-galactosidase activity (Figure 1). This discovery underscores the necessity of recognizing the impact of environmental factors in instigating cardiac aging processes.

Oxidative stress and DNA double-strand breaks are well-known contributors to cellular senescence and cardiac aging [16,37]. It has been documented that PM2.5 exposure can lead to oxidative stress, with one of its significant consequences being DNA damage [38]. Here, we observed that EOM exposure evoked ROS, mtROS overproduction, and DNA damage in H9c2 cardiomyoblasts. These observations are consistent with studies in mice and our previous findings in zebrafish [5,39]. Interestingly, we also discovered that the levels of ROS, mtROS, and DNA damage were significantly reduced when the ROS scavenger NAC was added. Consequently, the use of NAC mitigated the senescence process triggered by PM2.5 in cardiomyoblasts (Figure 3). Taken together, our data provide insight into the potential therapeutic effects of regulating the redox state in the context of PM2.5-induced cardiac senescence.

Our present investigation unveiled the activation of the AhR signaling pathway in H9c2 cardiomyoblasts following EOM exposure. Within our H9c2 culture system, we observed an upregulation in p21 and p16 expression levels upon exposure to EOM, an effect notably diminished by the introduction of the AhR antagonist CH. While prior research has extensively documented the involvement of the AhR signaling pathway in skin aging [40,41], the precise mechanism through which AhR modulates cellular senescence remains elusive. Notably, through bioinformatics analysis, we identified an AhR binding site within the promoter region of p16 (Supplementary Figure S1). Consequently, we postulated that AhR potentially functions as a direct transcriptional regulator of p16 expression. Furthermore, our results demonstrated that CH administration attenuated the excessive generation of ROS/mtROS and prevented the DNA damage induced by EOM. This highlights the significant contribution of AhR-mediated ROS overproduction in PM2.5-induced cardiac senescence (Figure 4 and Figure 5).

## 5. Conclusions

**Figure 6:**
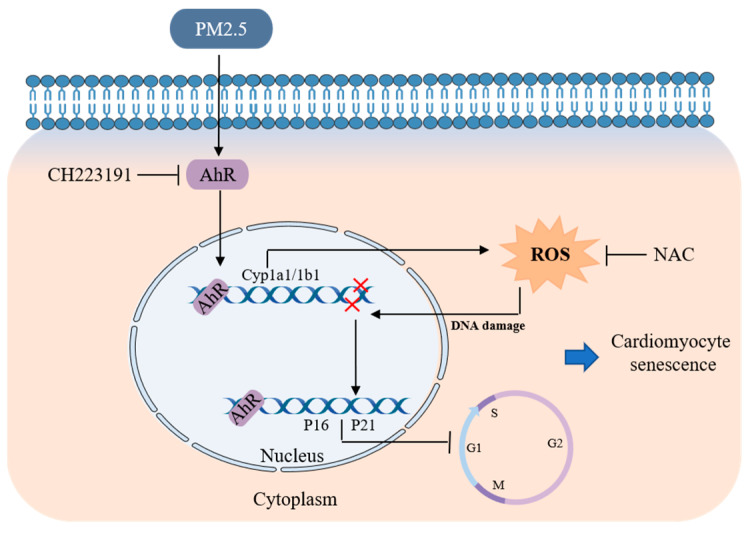
Proposed mechanism underlying the EOM-induced cardiac senescence. Activation of AhR by EOM triggers an excessive generation of ROS, consequently culminating in DNA damage, cell cycle arrest, and the subsequent induction of senescence in cardiomyoblasts. (CH223191: AhR antagonist; NAC: ROS scavenger).

Our results demonstrated that AhR, activated by EOM, induces ROS overproduction, leading to DNA damage and cell cycle arrest, ultimately resulting in cardiomyoblast senescence (Figure 6). Given the prevalence of AhR antagonists and ROS scavengers in food and clinical drugs, our study holds promise for combating PM2.5-induced cardiac aging. Further investigation into the in vivo effects of the AhR/ROS axis on cardiac aging caused by PM2.5 would be particularly useful.

## Data Availability

Data are available upon request.

## Supplementary Materials

The following supporting information can be downloaded at: https://www.mdpi.com/article/10.3390/antiox13070786/s1, Table S1: Primers used in this study. Figure S1: The predicted AhR binding sites on the p16 gene, with the red marking indicating the potential binding site of AhR in the promoter region of the p16 gene.

## References

[1] Feng S., Gao D., Liao F., Zhou F., Wang X. (2016). The health effects of ambient PM2.5 and potential mechanisms. Ecotoxicol. Environ. Saf..

[2] World Health Organization (2021). Particulate Matter (PM(2.5) and PM(10)), Ozone, Nitrogen Dioxide, Sulfur Dioxide and Carbon Monoxide.

[3] Fushimi A., Nakajima D., Furuyama A., Suzuki G., Ito T., Sato K., Fujitani Y., Kondo Y., Yoshino A., Ramasamy S. (2021). Source contributions to multiple toxic potentials of atmospheric organic aerosols. Sci. Total Environ..

[4] Yue D., Zhang Q., Zhang J., Liu W., Chen L., Wang M., Li R., Qin S., Song X., Ji Y. (2023). Diesel exhaust PM2.5 greatly deteriorates fibrosis process in pre-existing pulmonary fibrosis via ferroptosis. Environ. Int..

[5] Ji C., Tao Y., Li X., Wang J., Chen J., Aniagu S., Jiang Y., Chen T. (2023). AHR-mediated m(6)A RNA methylation contributes to PM(2.5)-induced cardiac malformations in zebrafish larvae. J. Hazard. Mater..

[6] Costantino S., Paneni F., Cosentino F. (2016). Ageing, metabolism and cardiovascular disease. J. Physiol..

[7] Mordukhovich I., Coull B., Kloog I., Koutrakis P., Vokonas P., Schwartz J. (2015). Exposure to sub-chronic and long-term particulate air pollution and heart rate variability in an elderly cohort: The Normative Aging Study. Environ. Health.

[8] Peralta A.A., Schwartz J., Gold D.R., Coull B., Koutrakis P. (2021). Associations between acute and long-term exposure to PM2.5 components and temperature with QT interval length in the VA Normative Aging Study. Eur. J. Prev. Cardiol..

[9] Henneberger A., Zareba W., Ibald-Mulli A., Rückerl R., Cyrys J., Couderc J.P., Mykins B., Woelke G., Wichmann H.E., Peters A. (2005). Repolarization changes induced by air pollution in ischemic heart disease patients. Environ. Health Perspect..

[10] Schikowski T., Hüls A. (2020). Air Pollution and Skin Aging. Curr. Environ. Health Rep..

[11] Wilker E.H., Preis S.R., Beiser A.S., Wolf P.A., Au R., Kloog I., Li W., Schwartz J., Koutrakis P., DeCarli C. (2015). Long-term exposure to fine particulate matter, residential proximity to major roads and measures of brain structure. Stroke.

[12] Gude N.A., Broughton K.M., Firouzi F., Sussman M.A. (2018). Cardiac ageing: Extrinsic and intrinsic factors in cellular renewal and senescence. Nat. Rev. Cardiol..

[13] Chen M.S., Lee R.T., Garbern J.C. (2022). Senescence mechanisms and targets in the heart. Cardiovasc. Res..

[14] Chadda K.R., Ajijola O.A., Vaseghi M., Shivkumar K., Huang C.L., Jeevaratnam K. (2018). Ageing, the autonomic nervous system and arrhythmia: From brain to heart. Ageing Res. Rev..

[15] Wang Q., Gan X., Li F., Chen Y., Fu W., Zhu X., Xu D., Long M., Xu D. (2019). PM(2.5) Exposure Induces More Serious Apoptosis of Cardiomyocytes Mediated by Caspase3 through JNK/P53 Pathway in Hyperlipidemic Rats. Int. J. Biol. Sci..

[16] López-Otín C., Blasco M.A., Partridge L., Serrano M., Kroemer G. (2023). Hallmarks of aging: An expanding universe. Cell.

[17] Ren F., Ji C., Huang Y., Aniagu S., Jiang Y., Chen T. (2020). AHR-mediated ROS production contributes to the cardiac developmental toxicity of PM2.5 in zebrafish embryos. Sci. Total Environ..

[18] Denison M.S., Nagy S.R. (2003). Activation of the aryl hydrocarbon receptor by structurally diverse exogenous and endogenous chemicals. Annu. Rev. Pharmacol. Toxicol..

[19] Panda S.K., Peng V., Sudan R., Ulezko Antonova A., Di Luccia B., Ohara T.E., Fachi J.L., Grajales-Reyes G.E., Jaeger N., Trsan T. (2023). Repression of the aryl-hydrocarbon receptor prevents oxidative stress and ferroptosis of intestinal intraepithelial lymphocytes. Immunity.

[20] Siti H.N., Kamisah Y., Kamsiah J. (2015). The role of oxidative stress, antioxidants and vascular inflammation in cardiovascular disease (a review). Vascul. Pharmacol..

[21] Guo Y., Guan T., Shafiq K., Yu Q., Jiao X., Na D., Li M., Zhang G., Kong J. (2023). Mitochondrial dysfunction in aging. Ageing Res. Rev..

[22] Bernard M., Yang B., Migneault F., Turgeon J., Dieudé M., Olivier M.A., Cardin G.B., El-Diwany M., Underwood K., Rodier F. (2020). Autophagy drives fibroblast senescence through MTORC2 regulation. Autophagy.

[23] Vogel C.F.A., Van Winkle L.S., Esser C., Haarmann-Stemmann T. (2020). The aryl hydrocarbon receptor as a target of environmental stressors—Implications for pollution mediated stress and inflammatory responses. Redox Biol..

[24] Chen J., Zhang M., Zou H., Aniagu S., Jiang Y., Chen T. (2023). PM2.5 induces mitochondrial dysfunction via AHR-mediated cyp1a1 overexpression during zebrafish heart development. Toxicology.

[25] Fu B., Chen T., Jiang B., Feng H., Zhu Z., Li M., Zhang G., Jiang Y. (2024). 6PPDQ induces cardiomyocyte senescence via AhR/ROS-mediated autophagic flux blockage. Environ. Pollut..

[26] Teng Z., Jiang B., Wang J., Liu T., Aniagu S., Zhu Z., Chen T., Jiang Y. (2023). Regulation of Cx43 and its role in trichloroethylene-induced cardiac toxicity in H9C2 rat cardiomyocytes. Chemosphere.

[27] North B.J., Sinclair D.A. (2012). The intersection between aging and cardiovascular disease. Circ. Res..

[28] Brook R.D., Rajagopalan S., Pope C.A., Brook J.R., Bhatnagar A., Diez-Roux A.V., Holguin F., Hong Y., Luepker R.V., Mittleman M.A. (2010). Particulate matter air pollution and cardiovascular disease: An update to the scientific statement from the American Heart Association. Circulation.

[29] Yao Y., Lv X., Qiu C., Li J., Wu X., Zhang H., Yue D., Liu K., Eshak E.S. (2022). The effect of China’s Clean Air Act on cognitive function in older adults: A population-based, quasi-experimental study. Lancet Healthy Longev..

[30] Gao H., Nepovimova E., Heger Z., Valko M., Wu Q., Kuca K., Adam V. (2023). Role of hypoxia in cellular senescence. Pharmacol. Res..

[31] Nwanaji-Enwerem J.C., Cardenas A., Chai P.R., Weisskopf M.G., Baccarelli A.A., Boyer E.W. (2019). Relationships of Long-Term Smoking and Moist Snuff Consumption With a DNA Methylation Age Relevant Smoking Index: An Analysis in Buccal Cells. Nicotine Tob. Res..

[32] Zhou Z., Shao T., Qin M., Miao X., Chang Y., Sheng W., Wu F., Yu Y. (2018). The effects of autophagy on vascular endothelial cells induced by airborne PM2.5. J. Environ. Sci..

[33] Yuan X., Wang Y., Li L., Zhou W., Tian D., Lu C., Yu S., Zhao J., Peng S. (2016). PM(2.5) induces embryonic growth retardation: Potential involvement of ROS-MAPKs-apoptosis and G0/G1 arrest pathways. Environ. Toxicol..

[34] Meng M., Jia R., Wei M., Meng X., Zhang X., Du R., Sun W., Wang L., Song L. (2022). Oxidative stress activates Ryr2-Ca(2+) and apoptosis to promote PM(2.5)-induced heart injury of hyperlipidemia mice. Ecotoxicol. Environ. Saf..

[35] He S., Sharpless N.E. (2017). Senescence in Health and Disease. Cell.

[36] Hu C., Zhang X., Teng T., Ma Z.G., Tang Q.Z. (2022). Cellular Senescence in Cardiovascular Diseases: A Systematic Review. Aging. Dis..

[37] Puente B.N., Kimura W., Muralidhar S.A., Moon J., Amatruda J.F., Phelps K.L., Grinsfelder D., Rothermel B.A., Chen R., Garcia J.A. (2014). The oxygen-rich postnatal environment induces cardiomyocyte cell-cycle arrest through DNA damage response. Cell.

[38] Wu J., Shi Y., Asweto C.O., Feng L., Yang X., Zhang Y., Hu H., Duan J., Sun Z. (2017). Fine particle matters induce DNA damage and G2/M cell cycle arrest in human bronchial epithelial BEAS-2B cells. Environ. Sci. Pollut. Res. Int..

[39] Fan X., Dong T., Yan K., Ci X., Peng L. (2023). PM2.5 increases susceptibility to acute exacerbation of COPD via NOX4/Nrf2 redox imbalance-mediated mitophagy. Redox Biol..

[40] Shi Y., Zeng Z., Liu J., Pi Z., Zou P., Deng Q., Ma X., Qiao F., Xiong W., Zhou C. (2021). Particulate matter promotes hyperpigmentation via AhR/MAPK signaling activation and by increasing α-MSH paracrine levels in keratinocytes. Environ. Pollut..

[41] Vogeley C., Esser C., Tüting T., Krutmann J., Haarmann-Stemmann T. (2019). Role of the Aryl Hydrocarbon Receptor in Environmentally Induced Skin Aging and Skin Carcinogenesis. Int. J. Mol. Sci..

