# Supplementary material for: PM2.5 Induces Cardiomyoblast Senescence via AhR-Mediated Oxidative Stress
Source: Antioxidants (Basel). 2024 Jun 28;13(7):786. doi: 10.3390/antiox13070786 (PMC11274155; doi:10.3390/antiox13070786)

Table S1. Primers used in this study.

Genes	Sense (5'-3')	Antisense (5'-3')
GAPDH	GACATGCCGCCTGGAGAAAC	AGCCCAGGATGCCCTTTAGT
Cyp1b1	CAGGCAGAATTGGATCAGGTCGTG	GTGGTGGCATGAGGAATAGTGACAG
Cyp1a1	TTCGCTACCTACCCAACCCTTCC	TTTGTAGTGCTCCTTGACCATCTTCTG
P16	TGCGGTATTTGCGGTATCTACTCTC	TAGTCTCGCGTTGCCAGAAGTG
P21	AGCAGTTGAGCCGCGATTG	ACCCAGGGCTCAGGTAGATCTTG

Figure S1. The predicted AhR binding sites on the p16 gene, with the red marking indicating the potential binding site of AhR in the promoter region of the p16 gene.

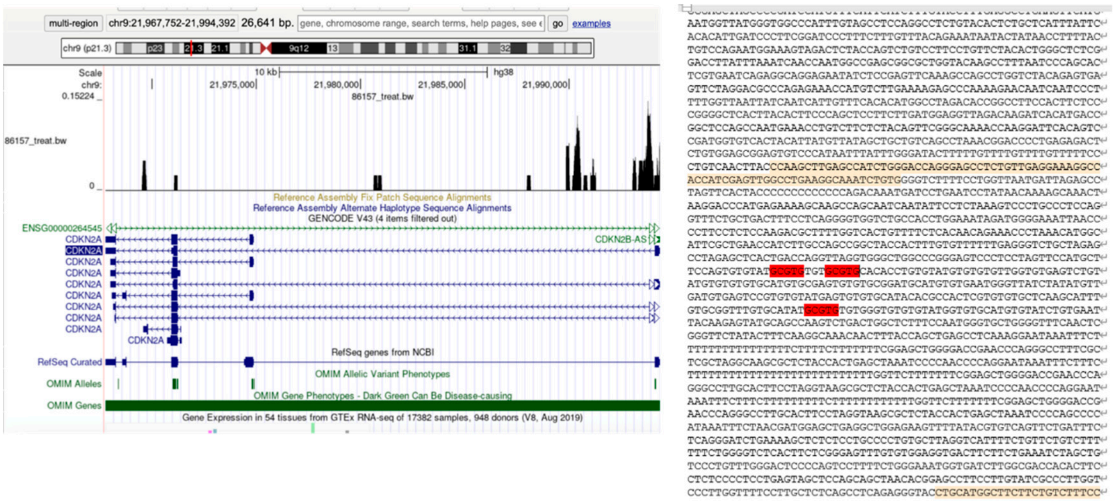

Supplement: Supplementary file 1 [file antioxidants-13-00786-s001.zip › antioxidants-3036294-supplementary.pdf]
